# Changes in fruit and vegetable consumption during the transition to parenthood: longitudinal evidence from Australia and the United Kingdom

**DOI:** 10.3389/fpubh.2025.1673209

**Published:** 2026-01-13

**Authors:** Silvan Munschek, Philipp Linden, Nadine Reibling

**Affiliations:** Faculty of Health Sciences, Fulda University of Applied Sciences, Fulda, Germany

**Keywords:** comparative longitudinal analysis, dietary behavior, difference-in-difference, fruit and vegetable, parenthood, transition

## Abstract

**Background:**

Families are an important context for healthy eating. This longitudinal study investigates how becoming a parent affects the fruit and vegetable consumption of men and women.

**Methods:**

This study uses two harmonized nationally representative longitudinal household surveys for Australia (*N* = 2,288 women and 2,479 men) and the United Kingdom (*N* = 5,424 women and 4,275 men) with data collected between 2007 and 2018. Changes in fruit and vegetable consumption are studied from three and more years before the birth of the first child until 6 years and more after birth using a difference-in-difference design.

**Results:**

The transition to parenthood increases the fruit and vegetable consumption in Australia with a significant increase in the proportion of men and women consuming at least one portion of fruit or vegetables per day. While for Australian mothers, this change is visible already 1–2 years before birth, for fathers the change occurs postnatally. The effect extends over several years after birth. For Australian mothers, the increase is due exclusively to an increase in the consumption of fruit, while for men small effects are found for both fruits and vegetables. Additionally, the increase in fruit and vegetable consumption is more pronounced among highly educated parents. There are no significant changes in the daily consumption of fruit and vegetables with parenthood in the United Kingdom.

**Conclusion:**

Individuals’ fruit and vegetable consumption is not strongly affected by becoming a parent. When it is, the effect is positive for both men and women, and greater for those with higher levels of education. Differences between countries indicate the importance of cultural contexts in the influence of parenthood on diet.

## 1 Introduction

A healthy diet, particularly the regular consumption of fruits and vegetables, is a cornerstone of healthy living and is emphasized in dietary guidelines worldwide (1, 2). Fruit and vegetable intake is a central dietary recommendation and is commonly used as a proxy for overall diet quality, as it reflects broader dietary patterns characterized by higher nutrient density and lower intake of energy-dense foods (3, 4). It is also an important determinant of long-term health. Analyses of the global burden of disease have identified low fruit and vegetable consumption as a primary dietary risk factor for years of life lost (YLL) and disability-adjusted life years (DALYs) (5). Furthermore, both higher and more diverse fruit and vegetable intake have been linked to greater nutrient adequacy, higher overall diet quality, and healthier lifestyle patterns (6, 7). Research has also demonstrated that patterns of fruit and vegetable consumption undergo changes during major life-course transitions, reflecting broader adjustments to lifestyle and health behaviors (8).

Understanding how dietary habits change during significant life transitions, such as parenthood, is essential given their long-term public health implications and parents’ critical role in shaping their children’s eating behaviors (8, 9). Parenthood introduces substantial lifestyle shifts that can influence dietary behaviors positively or negatively due to new demands, responsibilities, and altered role expectations (10–13). On the one hand, parenthood may encourage fruit and vegetable consumption as parents assume greater responsibility, adhere to societal expectations of “good parenting,” and seek to serve as positive role models for their children. On the other hand, the demands of parenthood – such as time pressure, increased caregiving responsibilities, and fatigue – can hinder the preparation and consumption of meals rich in fruits and vegetables (11–13). Existing quantitative research exploring the relationship between parenthood and fruit and vegetable consumption has yielded inconsistent results. Cross-sectional studies report mixed outcomes: some indicate increased consumption among parents, while others find decreases (14). Similarly, longitudinal studies (see Supplementary Table A1) have not reached conclusive results. Some studies identify dietary improvements following parenthood, others report no significant changes, and yet others suggest declines in healthy eating behaviors (15–20). These investigations frequently face methodological constraints, including short observation periods and inadequate control for unobserved individual factors such as personality traits, potentially biasing the observed relationship (21–24).

To address these limitations, this study investigates the impact of becoming a parent on long-term trajectories of fruit and vegetable consumption among mothers and fathers, using extended observation periods of up to 7 years in the United Kingdom (UK) and up to 10 years in Australia (AU). By employing a difference-in-difference approach, we account for time-invariant unobserved heterogeneity, thereby ensuring a more robust estimation of the effects of parenthood. Moreover, acknowledging the diverse experiences of parenthood, this study separately examines the effects on mothers and fathers and across different educational groups, reflecting socioeconomic variations in parental resources and behaviors (25, 26).

Additionally, this research incorporates a cross-national comparative perspective by analyzing harmonized data from Australia and the United Kingdom, both classified as liberal welfare states characterized by market-oriented policies and means-tested or non-universal family benefits. This allows us to examine whether the relationship between parenthood and dietary habits is consistent within welfare regimes sharing similar characteristics (27, 28). Thus, the study seeks to clarify whether previously inconsistent findings can be attributed to country-specific factors or if they represent broader patterns.

## 2 Materials and methods

### 2.1 Data basis

For the present study, a harmonized data set from the longitudinal household surveys “Household, Income and Labour Dynamics” (HILDA: (29)) for AU and the “UK Household Longitudinal Study” for the UK (UKHLS: (30)) was created. Australia and the United Kingdom, which are both considered liberal welfare states, were selected primarily because of the availability of high-quality longitudinal panel data with information on dietary outcomes, which are essential for a difference-in-difference approach. For the analyses, the data set was restricted exclusively to individuals who were born between 1970 and 1991, who resided in the countries under study between 2007 and 2017 for the Australian data and between 2011 and 2018 for the UK data, and who were at least 18 years of age. By implementing this restriction, we ensured that the sample comprised individuals who were at an age typical for starting a family during the observation period. This is important to construct comparable samples of childless persons and parents. To observe changes over time, the sample was further restricted to respondents with no missing values on the model variables and who were observed at least once before the birth of their first child (*N* = 34,867). The results presented in Table 1 demonstrate the number of cases for both men and women across selected countries and observational years, while ensuring the absence of missing values on the specified model variables.

**Table 1:** Case numbers over countries for observed individuals, transitions to parenthood, observational periods, and observational years, stratified by gender.

Dataset	Observed individuals		Transitions to…		Observational years		Observational period
	Women	Men	Motherhood	Fatherhood	Women	Men	
UKHLS	5,424	4,275	322	239	13,159	9,906	2011, 2014, 2016, 2018
HILDA	2,288	2,479	220	194	5,746	6,056	2007, 2009, 2013, 2017

Source: Harmonized dataset based on HILDA and UKHLS panel data, *N* = 34,867 person-years. Own compilation and depiction.

### 2.2 Variables

The WHO has set a recommendation of at least five portions of fruit and vegetables per day (2). However, due to the varying questions and answer categories used in the two datasets, we had to construct a simpler measure to harmonize the data (see Table 2). Thus, our dependent variable measures whether the respondents consumed fruit at least once per day (1) or not (0) and vegetables at least once per day (1) or not (0), and a combined measure that reflects the daily consumption of at least one food group - daily consumption of fruit and/or vegetables (1) or not (0).

**Table 2:** Items and answer categories of the diet survey measures, and harmonized variables used for the analysis.

Dependent variable		Frequency of weekly fruit consumption			Frequency of weekly vegetable consumption		
		Question	Answer Categories	Harmonized	Question	Answer Categories	Harmonized
Survey	UKHLS	“Including tinned, frozen, dried and fresh fruit, on how many days in a usual week do you eat fruit?”	“Never,” “1–3 days,” “4–6 days,” “every day” and “refusal / do not know/ proxy”	(0) Non-daily fruit consumption: Never, 1–3 days, 4–6 days; (1) Daily fruit consumption: every day	“Including tinned, frozen and fresh vegetables, on how many days in a usual week do you eat vegetables? Do not include potatoes, crisps or chips.”	“Never,” “1–3 days,” “4–6 days,” “every day” and “refusal / do not know/ proxy”	(0) Non-daily vegetable consumption: Never, 1–3 days, 4–6 days; (1) Daily vegetable consumption: every day
	HILDA	“Including tinned, frozen, dried and fresh fruit, on how many days in a usual week do you eat fruit?”	“Do not eat fruit in a usual week”, “1 day per week”, “2 day per week” […] “7 days per week” and “Refused/Not stated”	(0) Non-daily fruit consumption: 1–6 days per week; (1) Daily fruit consumption: 7 days per week	“Including tinned, frozen and fresh vegetables, on how many days in a usual week do you eat vegetables?”	“Do not eat vegetable in a usual week” “1 day per week”, “2 days per week” […] “7 days per week” and “Refused/Not stated”	(0) Non-daily vegetable consumption: 1–6 days per week; (1) Daily vegetable consumption: 7 days per week

To estimate the parenthood effect, we operationalize the transition to parenthood via two constructed variables. First, a time-sensitive parenthood indicator, which measures the time before/after parenthood through a categorical variable: 3 years and more (*BY-3a+*) or 1 and 2 years before birth (*BY-1/2a*) and 1 year (*BY+1a*), 2 to 3 years (*BY+2/3a*), 4 to 5 (*BY+4/5a*) and 5 years and more (*BY+6+*) after birth. Observations of individuals who remain childless during the whole observation period are merged with the category BY-3a + and form the reference category in our analyses. Second, a time-constant parenthood indicator, which takes a value of 1 if individuals are ever observed as a parent or 0 if they were not. It captures time-constant differences in the outcomes between parents and non-parents that might otherwise distorts the coefficients of the time-varying parenthood variable.

Moreover, we controlled for variables that may influence diet, but are exogenous to parenthood: metric age, family structure (*living without or with a partner*) and education with two levels. Primary/Secondary level education thereby reflected ISCED level 0–4, from early childhood education up to preparatory courses for attending a university or educational programs that prepare for direct entry into the labor market, whereas tertiary level education reflected ISCED level 5–8, from higher vocational training, technician or community college up to doctoral degree. Finally, to incorporate time trends into the model, we controlled for the birth cohort (*1970–1979 vs. 1980–1991*), and year of survey (*2007–2011 for Australia and 2007–2017 for UK at 4 timepoints each*). Because some of the measurements differed by country, we used the harmonized versions of the variables provided in the Comparative Panel File by Turek, Kalmijn and Leopold (31).

### 2.3 Statistical analysis

This study focuses on the overall effect of parenthood on three dietary outcomes: (1) daily fruit consumption, (2) daily vegetable consumption, and (3) daily consumption of either fruit and/or vegetables. These binary outcomes are analyzed separately for women and men who typically become parents. This effect is also referred to as the Average Treatment Effect on the Treated (ATT). We define the ATT as the difference in the share of daily fruit, or vegetable consumption or a combined measure of both (Y = 1) for those, who have become parent (X = 1), between their observed behavior as parents and the hypothetical behavior if they had remained childless (X = 0) (Equation 1):


$$\mathrm{ATT}=P[Y^{x=1}=1|X=1]-P[Y^{x=0}=1|X=1]$$ (1)

The probability of each outcome under the causal condition of parenthood can be estimated by $P[Y^{x=1}=1|X=1]$ , which is the observed probability for parents. It is not possible to estimate the second probability $P[Y^{x=0}=1|X=1]$ , that is, the probability if the parents had not become parents. Therefore, we call this second probability a counterfactual probability. Nevertheless, we can make certain assumption to estimate this counterfactual probability. The assumptions are not arbitrary but are derived from observations of childless people or observations of parents before the transition to parenthood.

If we assume that there are no *unmeasured* differences between childless individuals and parents that affect the daily fruit or vegetable consumption other than parenthood itself, we could simply use the observations in the outcomes of childless individuals to estimate the ATT within a simple pooled OLS regression model. *Measured* differences can thereby be incorporated through the integration of control variables (e.g., level of education). Yet, there are several reasons to doubt this assumption of no time unmeasured time-constant heterogeneity between parent and non-parents. For example, stable preferences and life goals not captured by the control variables could affect both the transition to parenthood and the level of daily fruit or vegetable consumption. To capture these group differences, we must extend the model specification to a model from the family of difference-in-difference models: a pooled OLS group fixed effects (POLS-GFE) regression model (32). It assumes that childless people and parents follow a parallel trend in the probability of daily fruit or vegetable consumption. We model this assumption by including the time-constant parenthood indicator that captures whether a respondent is ever observed as parent or remains childless throughout the observation period (Equation 2):


$$P[Y_{t}=1|X_{t},Z_{t}]=\delta_{0}+\delta_{1}x_{t}+\delta_{2}x+\delta_{k}z_{k}$$ (2)

In this equation, Y_t_ mirrors the indicator of daily fruit and vegetable consumption on the time-varying parenting variable X_t_, while Z reflects measured controls. The regression coefficient *δ*_2_ then captures the time-constant difference in the probability of the respective dietary outcome between parents and childless individuals that is not already captured by the control variables Z, whereas $\delta_{k}$ captures all differences in the control variables that may vary over time. If the assumption hold (and the model is correctly specified), $\beta_{1}$ identifies the ATT of interest.

In the analysis, the three dietary outcomes are estimated separately for both countries and stratified by sex. Furthermore, comparisons are conducted between educational groups to investigate how the parenthood effect varies across socioeconomic position. For the implementation of the models, we applied a linear probability model based on a POLS throughout, despite the binary nature of the outcomes. The advantage of this specification is that OLS regression directly provides us with average marginal effects without further transformations, unlike nonlinear probability models and that individuals with no within-person variation can be retained in the OLS regression. Moreover, common problems of specifying a linear probability model with a binary outcome are not present in our data since the distribution of the outcome variable is relatively symmetrical and all independent variables except age are categorical, so that predictive values smaller than 0 and larger than 1 are practically excluded (33). Finally, we addressed potential heteroscedasticity by estimating all models with individual cluster-robust standard errors (31). To evaluate the validity of the parallel trends assumption in the difference-in-differences model, we examined pre-treatment trends in dietary outcomes among expectant and childless individuals. The corresponding plots (see Supplementary Figures A1–A3) show largely parallel developments in the years before the first birth, which supports the validity of this assumption.

## 3 Results

Table 3 shows the characteristics of our sample for AU and UK, broken down into respondents who never had children, parents before the birth of their first child and parents after the birth of their first child. The mean age of childless individuals in both countries lies between that of the pre-births and post-births groups. Parents before birth have the lowest mean age at 27.0 ± 4.9 in Australia and 31.0 ± 5.5 in the UK. The mean age of childless individuals in our sample is about 2 years higher in both countries (Australia: 29.0 ± 6.1 years, UK: 33.3 ± 7.5 years). As expected, those in the post-birth group are oldest (32.9 ± 4.9 years in Australia, 35.4 ± 5.4 years in the UK).

**Table 3:** Socio-demographic characteristics and dietary characteristics of Australian and British individuals who are always childless, parents pre-birth, and parents post-birth.

Characteristics	AU						UK					
	Always childless	Parents pre-birth	Parents post-birth	Always childless		Parents pre-birth		Parents post-birth	
	Mean±SD / %	*n*	Mean±SD/%	*n*	Mean±SD / %	*n*	Mean±SD / %	*n*	Mean±SD / %	*n*	Mean±SD / %	*n*
Demographic characteristic												
Age in year (Mean, SD)	29.0 ± 6.1	6,357	27.0 ± 4.9	2,795	32.9 ± 4.9	2,650	33.3 ± 7.5	17,253	31.0 ± 5.5	2,646	35.4 ± 5.4	3,166
Sex in %												
Female	45.5	2,856	52.7	1,488	53.1	1,402	55.3	9,730	58.4	1,559	58.4	1870
Male	54.5	3,501	47.3	1,307	46.9	1,248	44.7	7,523	41.6	1,087	41.6	1,296
Educational status in %												
Low/medium	56.2	3,630	57.6	1,635	51.1	1,387	51.8	8,540	46.5	1,196	43.7	1,358
High	43.8	2,727	42.4	1,160	48.9	1,263	48.2	8,713	53.5	1,450	56.3	1808
Partner status in %												
No partner	50.5	3,495	28.5	944	9.1	260	49.9	8,216	23.5	649	11.2	341
Partner	49.5	2,862	71.5	1851	90.9	2,390	50.1	9,037	76.5	1997	88.8	2,825
Birth cohort in %												
1970–1979	27.2	1998	29.3	760	29.1	900	45.9	8,840	37.9	955	37.8	1,286
1980–1991	72.8	4,359	70.7	2035	70.9	1750	54.1	8,413	62.1	1,691	62.2	1,880
Year of observation (AU / UK) in %												
2007/2011	20.3	1,291	37.9	1,058			31.6	5,450	59.4	1,572		
2009/2014	21.9	1,391	36.5	1,019	10.7	283	23.4	4,042	26.2	693	24.3	769
2013/2016	29.2	1853	25.7	718	32.6	865	24.1	4,156	14.4	381	33.7	1,067
2017/2018	28.7	1822			56.7	1,502	20.9	3,605			42.0	1,330
Diet variables												
Fruit and/or vegetable consumption in %												
Not daily	44.7	2,842	48.5	1,355	37	970	46.6	7,918	43.1	1,112	41.7	1,310
Daily	55.3	3,515	51.5	1,440	63	1,680	53.4	9,335	56.9	1,534	58.3	1856
Vegetable consumption in %												
Not daily	53.3	3,398	58.7	1,630	48.6	1,294	57.2	9,718	54.3	1,416	52.3	1,633
Daily	46.7	2,959	41.3	1,165	51.4	1,356	42.8	7,535	45.7	1,230	47.7	1,533
Fruit consumption in %												
Not daily	69	4,385	70.4	1959	60	1,585	64.2	11,054	60.9	1,574	59.2	1886
Daily	31	1972	29.6	836	40	1,065	35.8	6,199	39.1	1,072	40.8	1,280

Harmonized dataset based on HILDA and UKHLS panel data, *N* = 34,867 person-years. Own compilation and depiction, the percentage values are based on the aggregated proportions (mean value across all observations per person), while the mean age value represents the mean value per person across all survey waves to avoid distortions due to a varying number of data points per person.

There is a higher proportion of men in the always childless group (54.5% in AU, 44.7% in UK) compared with the values of the groups before and after birth. With respect to educational status, individuals with a higher level of education are more prominent in the post-birth group than in the childless group and the pre-birth group in both countries. In the UK a little more than half of the parents have a high level of education, while in Australia it is slightly less than half.

A notable disparity emerges in partnership status, with approximately half of the always childless individuals having a partner (49.5% in AU, 50.1% in UK), contrasted with the significantly higher proportion of individuals in partnerships within the pre-birth group (71.5% in AU, 76.5% in UK) and the post-birth group (90.9% in AU, 88.8% in UK).

In terms of dietary habits, only about half of the childless persons in both countries (55.3% in Australia and 53.4% in the UK) consumes fruits and/or vegetables on a daily basis. In all three groups a lower proportion of respondents consume fruits on a daily basis than vegetables. The proportion of individuals who report consumption is highest in the post-birth group for all three dependent variables in both countries. Compared to the pre-birth group, the daily consumption of vegetables (*t*(4,384) = −10.3290, *p* < 0.0001), fruit (*t*(4,384) = −10.1326, *p* < 0.0001), and fruit and/or vegetables (*t*(4,384) = −11.9877, *p* < 0.0001) is significantly higher among parents post-birth in AU. In the UK, the daily consumption of vegetables (*t*(7,562) = −3.3604, *p* < 0.0004), fruit (*t*(7,562) = −4.291 9, *p* < 0.0001) and fruit and/or vegetables (*t*(7,560) = −4.5663, *p* < 0.0001) is also significantly higher for parents post-birth compared with the pre-birth group, but the differences are smaller. However, while in Australia the pre-birth group has lower proportions of daily consumption of all three dietary variables than the childless, the opposite is observed in the UK.

### 3.1 The effect of parenthood on daily fruit and vegetable consumption over time and countries

**Figure 1:**
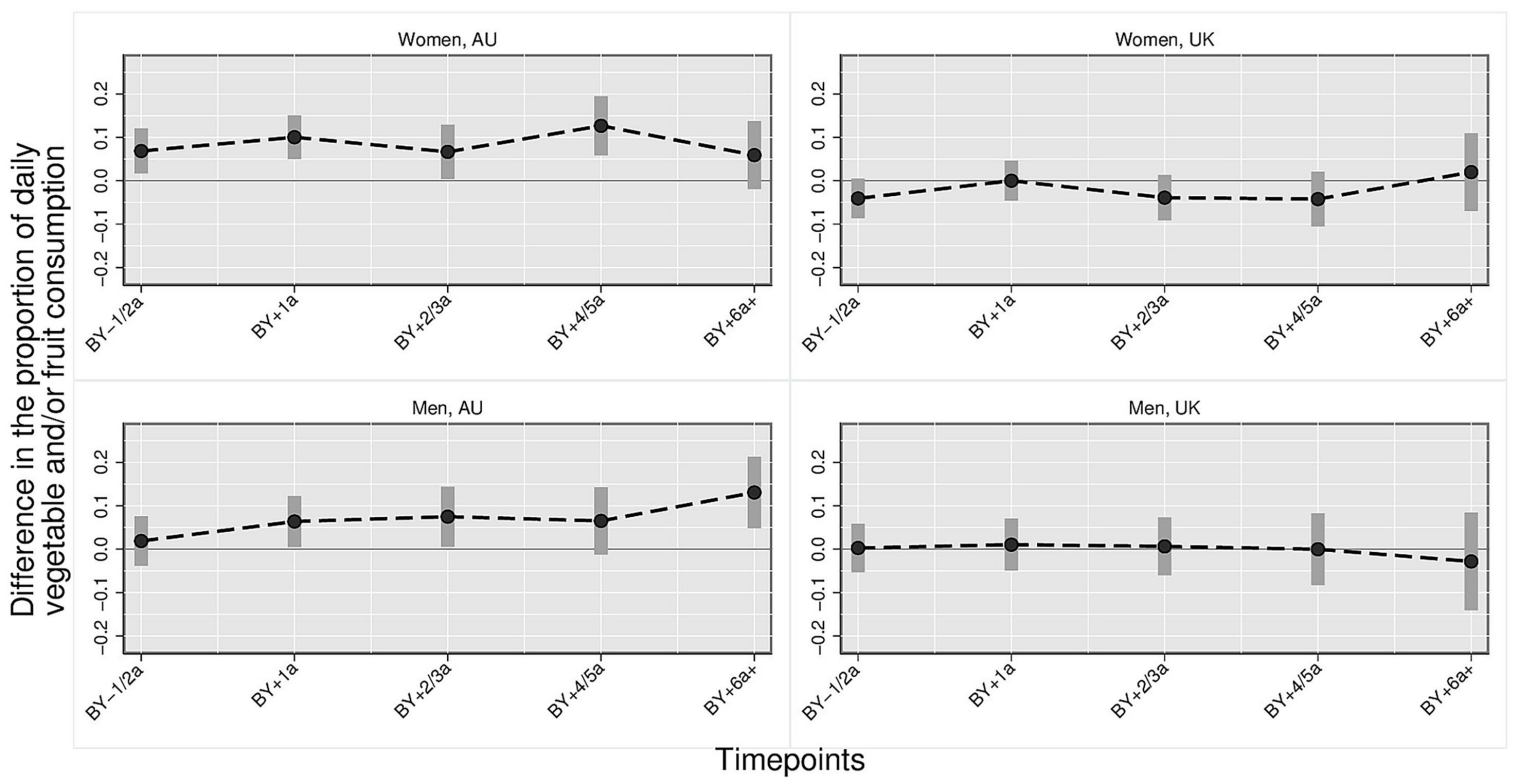
Daily fruit and/or vegetable consumption: parents vs. childless individuals across time/countries stratified by gender. Harmonized data set based on HILDA and UKHLS panel data, *N* = 34,867 person-years. Own compilation and depiction. Point estimates from multivariate fixed effects models controlling for age, education, partner status and birth cohort with 95% confidence interval based on robust standard errors. Key pattern: In AU, daily consumption of fruits and/or vegetables increases notably after the transition to parenthood, especially among mothers who already exhibit higher levels prior to birth. Among fathers, a smaller but steady increase is observed. In the UK, no significant differences appear over time.

**Figure 2:**
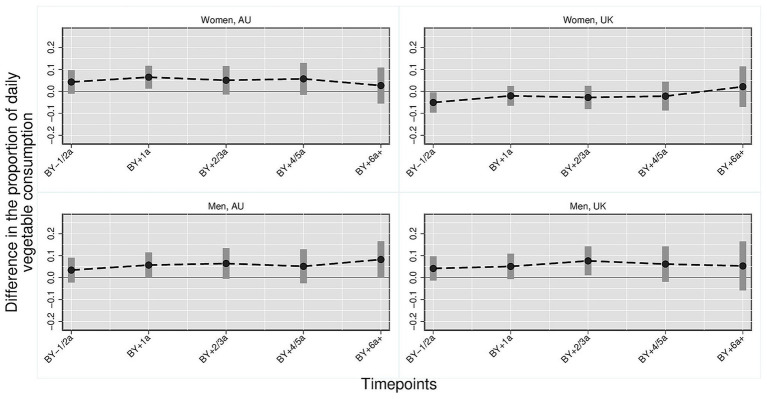
Daily vegetable consumption: parents vs. childless individuals across time and countries stratified by gender. Harmonized data set based on HILDA and UKHLS panel data, *N* = 34,867 person-years. Own compilation and depiction. Point estimates from multivariate fixed effects models controlling for age, education, partner status and birth cohort with 95% confidence interval based on robust standard errors. Key pattern: Differences in daily vegetable consumption are generally small. Among Australian mothers, a modest increase is visible 1 year after birth, whereas fathers show no significant changes. In the UK, women display a temporary decrease before birth and men a slight increase 2 to 3 years afterward.

**Figure 3:**
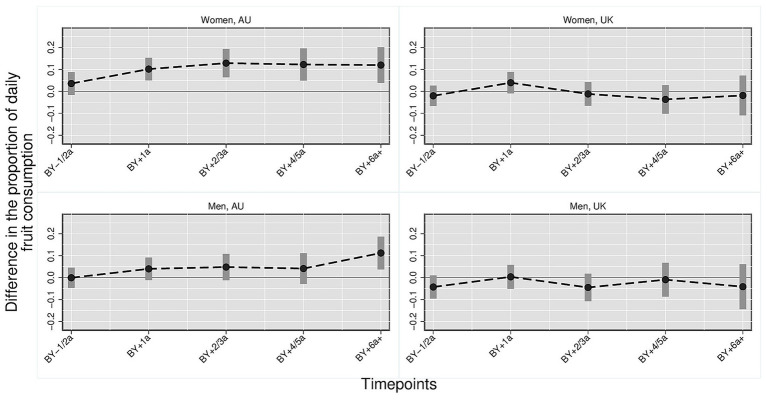
Daily fruit consumption: parents vs. childless individuals across time and countries stratified by gender. Harmonized data set based on HILDA and UKHLS panel data, *N* = 34,867 person-years. Own compilation and depiction. Point estimates from multivariate fixed effects models controlling for age, education, partner status and birth cohort with 95% confidence interval based on robust standard errors. Key pattern: In AU, daily fruit consumption increases notably among mothers and, to a lesser extent, among fathers, while in the UK no significant changes are observed across time points.

Figures 1–3 illustrate the trajectory of the effects of parenthood on dietary outcomes, stratified by gender. Based on the multivariate regression models, the figures display the estimated differences in the proportion of individuals who consume daily (1) fruits and/or vegetables, (2) vegetables and (3) fruits comparing parents at varying timepoints to the reference category which consists of childless persons and parents 3 year before the birth of the first child. In all figures, the *y*-axis represents the estimated mean differences in the proportion of individuals with the respective dietary outcomes relative to the reference category. The *x*-axis represents time relative to the transition to parenthood in categorical intervals (e.g., BY–1/2a = 1 to 2 years before birth; BY+1a = 1 year after birth). Each point indicates the point estimate derived from the regression models, and the vertical bars represent 95% confidence intervals. Differences were considered statistically significant if the confidence intervals did not cross the zero line. Positive values indicate higher proportions of individuals daily consuming the respective dietary outcome compared with the reference group, whereas negative values indicate lower proportions. Full regression tables with detailed model results are provided in the Supplementary Table A2.

*Daily fruit and/or vegetables* (Figure 1): The proportion of mothers and fathers who daily consume fruits and/or vegetables compared with childless individuals and mothers/fathers 3 years before birth is significantly higher in Australia, while there are no significant differences in the UK. In Australia, compared with the reference category, the proportion of mothers who consume fruit and/or vegetables daily is between 5 and 12 percentage points higher. This difference is already significant 1–2 years before birth (*anticipation effect*) (0.069, 95% CI [0.017, 0.121]) and continues to be significantly higher than in the reference category until 4/5 years (0.127, 95% CI [0.059, 0.195]) after birth but not beyond then. Among Australian men, the proportion of fathers who consume fruit and/or vegetables daily is significantly higher at one (0.064, 95% CI [0.005, 0.123]), two/three (0.075, 95% CI [0.006, 0.144]) years after birth and again at 6 years after birth (0.131, 95% CI [0.048, 0.213]) compared with childless men and men three or more years before birth. While not consistently statistically significant, the effect estimates were positive throughout the time after the transition to fatherhood in Australia.

*Daily vegetables* (Figure 2): Only small differences were found in the proportions of vegetable consumption between parents at varying time-points and childless individuals/parents 3 or more years before birth. In Australia, the proportion of mothers who daily eat vegetables is six percentage points higher 1 year after birth compared with the reference group (0.065, 95% CI [0.013, 0.117]). In contrast, fathers exhibited no significant disparities when compared to the reference group, however, effect estimates were consistently positive. The proportion of British women who consumed vegetables daily was five percentage points lower among women 1 to 2 years before childbirth compared with childless women and women three or more years before childbirth (−0.051, 95% CI [−0.097, −0.005]). Among British men, the proportion of fathers with daily vegetable consumption was about eight percentage points higher 2 to 3 years after becoming a father (0.076, 95% CI [0.010, 0.142]).

*Daily fruit* (Figure 3): The proportion of Australian mothers who consumed fruit daily was significantly higher than in the reference group from one till 6 years after birth. The difference ranges from 10.1 percentage points (0.101, 95% CI 0.050–0.153) to 12.9 percentage points (0.129, 95% CI [0.064, 0.194]). The proportion of fathers who daily consume fruits is also higher after birth than in the reference group. However, the difference is smaller and only significant six or more years after the birth of the first child. No significant differences for fruit consumption were found for the UK.

### 3.2 Education

**Figure 4:**
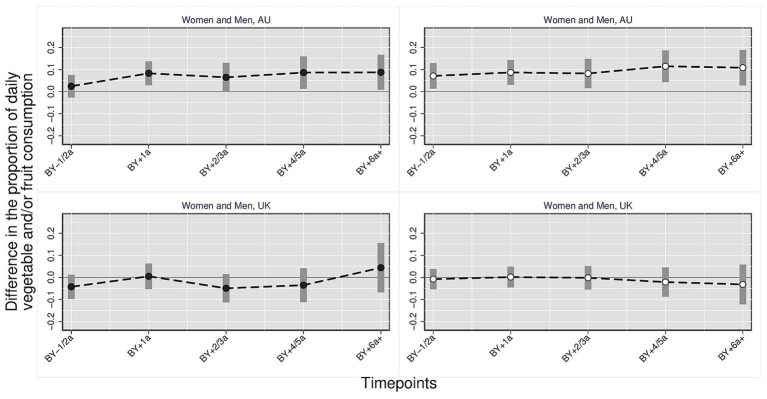
Daily fruit and/or vegetable consumption: parents vs. childless individuals across time and countries stratified by education. Black dots = medium/low level of education; White dots = high level of education. Harmonized data set based on HILDA panel data, *N* = 34,867 person-years. Own compilation and depiction. Point estimates from multivariate fixed effects models controlling for age, education, partner status and birth cohort with 95% confidence interval based on robust standard errors. Key pattern: In AU, parents with higher education show consistently higher levels of daily fruit and/or vegetable consumption, including before birth, while those with lower or medium education exhibit a smaller post-birth increase. In the UK, no significant differences appear across education groups.

**Figure 5:**
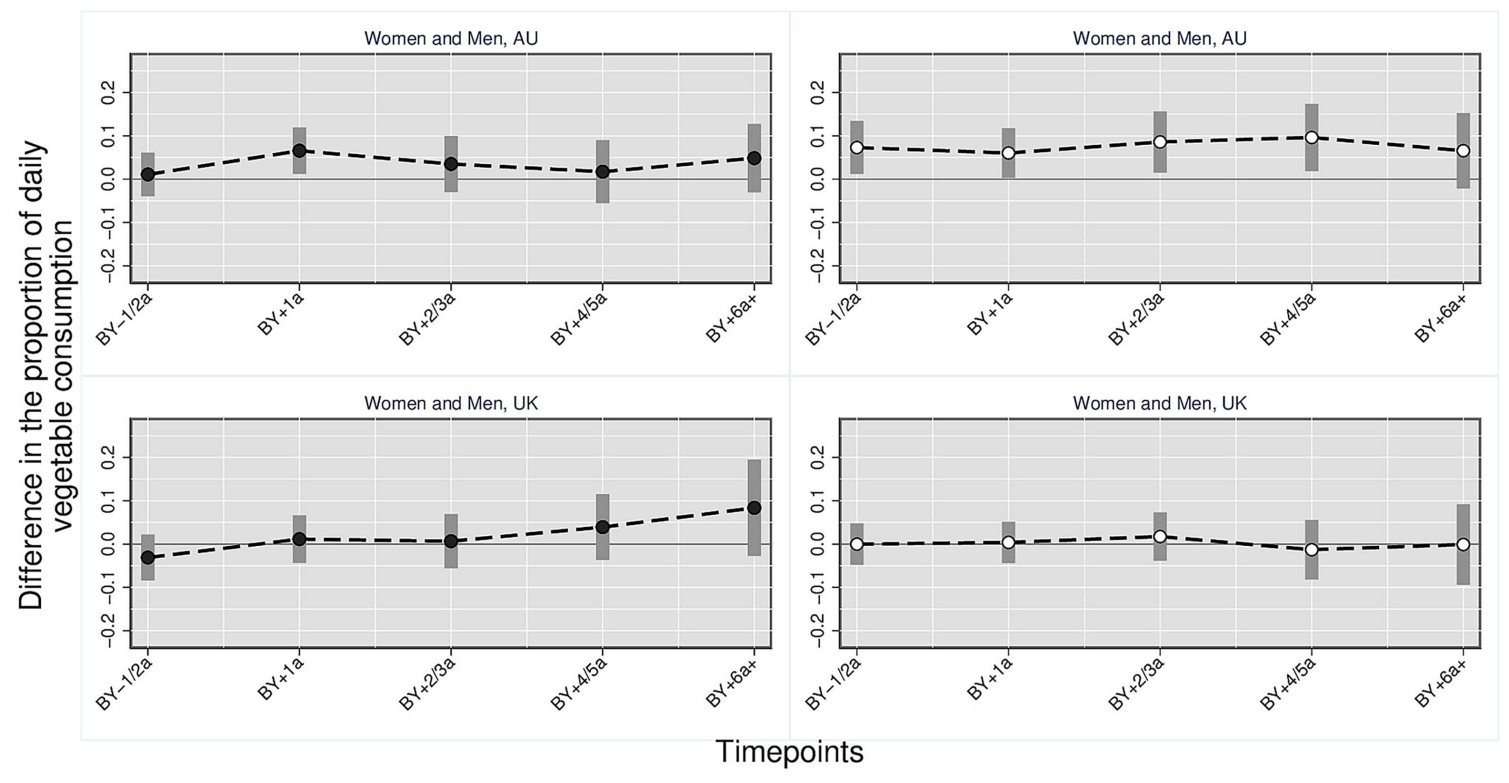
Daily vegetable consumption: parents vs. childless individuals across time and countries stratified by education. Black dots = medium/low level of education; White dots = high level of education. Harmonized data set based on HILDA panel data, *N* = 34,867 person-years. Own compilation and depiction. Point estimates from multivariate fixed effects models controlling for age, education, partner status and birth cohort with 95% confidence interval based on robust standard errors. Key pattern: In AU, parents with higher education show elevated levels of daily vegetable consumption before and after birth, while those with lower or medium education display a smaller increase following the transition to parenthood. In the UK, no significant differences are observed across education groups.

**Figure 6:**
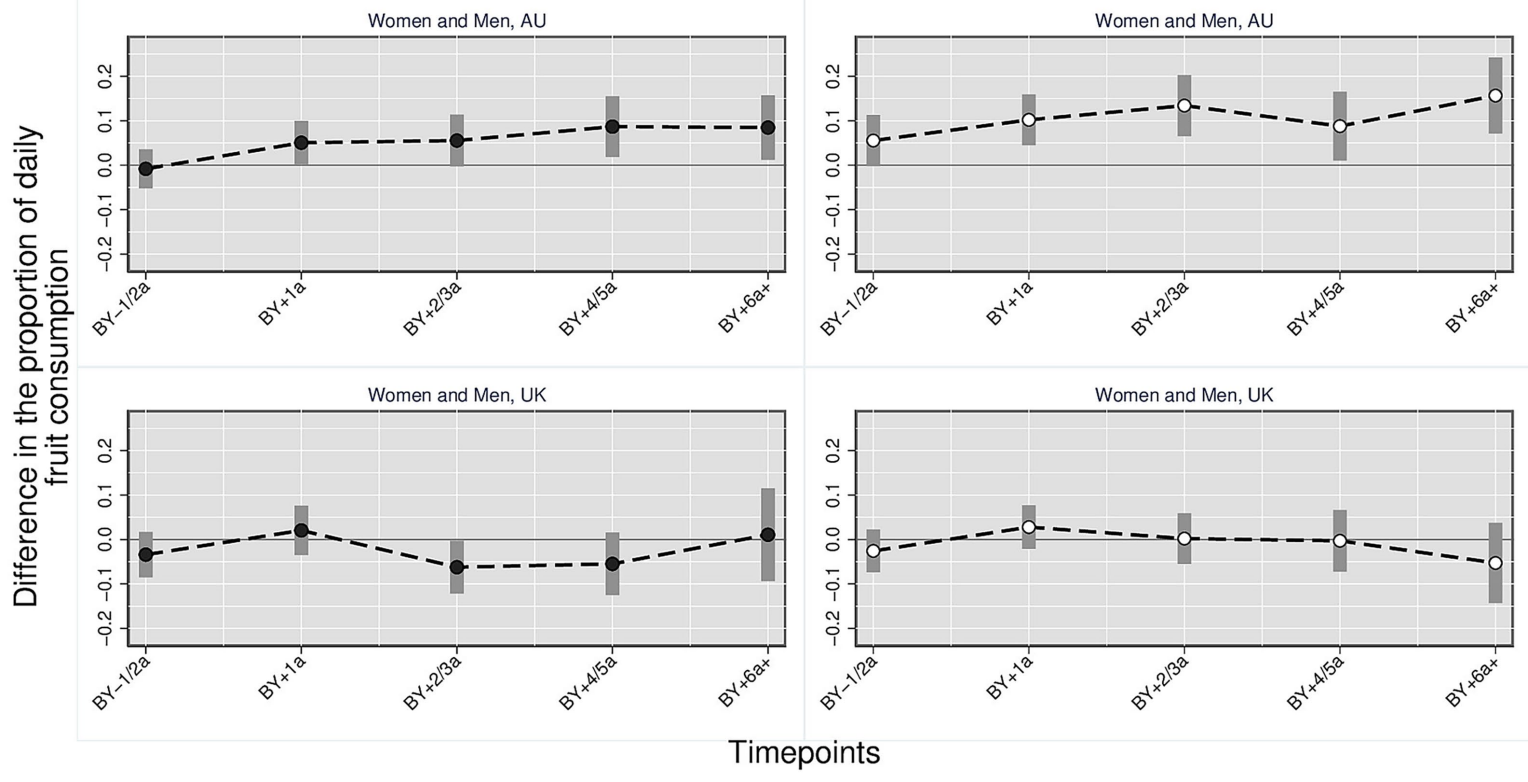
Daily fruit consumption: parents vs. childless individuals across time and countries stratified by education. Black dots = medium/low level of education; White dots = high level of education. Harmonized data set based on HILDA panel data, *N* = 34,867 person-years. Own compilation and depiction. Point estimates from multivariate fixed effects models controlling for age, education, partner status and birth cohort with 95% confidence interval based on robust standard errors. Key pattern: In AU, parents with higher education show consistently higher levels of daily fruit consumption after birth, while those with lower or medium education also display an overall increase across time. In the UK, daily fruit consumption remains largely stable, with a temporary lower level among parents with lower or medium education two to three years after birth.

Figures 4–6 show the effect of parenthood on dietary outcomes separately for individuals (women and men) with high and medium/low levels of education. Detailed regression results underlying these figures are presented in the Supplementary Table A3.

*Daily fruit and/or vegetables* (Figure 4): The proportion of Australian parents with a higher level of education who consume fruit and/or vegetables on a daily basis is already significantly higher before the birth (anticipation effect) (0.071, 95% CI [0.013, 0.129]) and remains consistently between 8 and 11 percentage points higher after the birth compared with childless people and parents three or more years before the birth. No significant anticipation effect can be observed in the medium/low education group. However, subsequent to the birth, a significant increase is observed in the proportion of parents in this group who consume fruit and/or vegetables daily. This increase is 8 to 9 percentage points compared to childless people/parents three or more years before the birth, with a short dip 2 to 3 years after the birth. In the UK, there is no significant difference in any of the education groups compared with the reference group.

*Daily vegetables* (Figure 5): In Australia, the proportion of parents with a medium/ low level of education who consume vegetables daily is around seven percentage points higher 1 year after birth compared to the reference group (0.066, 95% CI [0.012, 0.119]). The difference is statistically significant. For Australian parents with a higher level of education, the proportion who is eating vegetables daily is between 7 and 10 percentage points higher 1 to 2 years before birth (0.073, 95% CI [0.012, 0.134]) to 4 years after birth (0.096, 95% CI [0.018, 0.173]) compared to childless people/parents 3 years or more before birth. Within the United Kingdom, no significant disparities emerge among the education groups when parents are compared with the reference group.

*Daily fruit* (Figure 6): Regarding the daily consumption of fruit, parents with a higher level of education in Australia exhibit significantly higher proportions after birth compared to the reference group. The observed differences range from 8.8 (95% CI [0.010, 0.165]) to 15.7 (95% CI [0.071, 0.242]) percentage points, depending on the specified period. The group with a medium/low level of education exhibited higher proportions ranging from 5.1 (95% CI [0.002, 0.099]) to 8.7 (95% CI [0.019, 0.155]) percentage points compared to the reference group, with a slight decline observed 2 to 3 years following birth. In the UK, the proportion of daily fruit consumption is significantly lower in the medium/low-educated group 2 to 3 years after birth compared to the reference category (−0.062, 95% CI [−0.121, −0.003]). For all other time points, no significant differences were observed in the UK.

## 4 Discussion

In this study, we used harmonized panel data from the UK and Australia to investigate how parenthood affects fruit and vegetable consumption, paying careful attention to gender and educational differences as well as changes as children grow older. We theorized these relationships as the result of the conflicting role strain and role model implications associated with parenthood. Our methodological approach, consisting of general fixed-effects regression models provides robust estimations of changes in fruit and vegetable consumption over time.

Our main finding is that parenthood can increase fruit and vegetable consumption, but only very modestly, and the effect varies by food group, gender and country. First, an increased fruit and vegetable consumption due to parenthood was only visible in the Australian data, while in the UK (except for two point estimates) trajectories were not significantly different between parents and childless persons/ parents three or more years before birth. Second, the increase in fruit and vegetable consumption is larger for Australian women than for Australian men. Third, the larger benefits for Australian mothers (compared to fathers) are primarily due to the increase in the proportion of mothers who daily consume fruits which extends over the whole observation period up until six and more years after birth, while the association with vegetable consumption is less clear. Finally, we find a stronger increase in fruit and/or vegetable consumption among parents with a higher level of education than among parents with low or medium education. In the higher-educated group, a significant increase in fruit and vegetable consumption is already visible 1 to 2 years before birth and higher educated parents increase both their fruit and their vegetable consumption, while parents with low/ medium education mostly increase their fruit consumption. In the UK, an education-differentiated analysis shows again no significant differences.

When comparing our results with the existing studies with longer observation periods (≥1 year postpartum), they also find that dietary changes due to parenthood develop gradually. For instance, Elstgeest et al. (16) and Olson et al. (15) document sustained increases in fruit and/or vegetable intake among mothers in Australia and the U. S. over multiple years, aligning with our Australian data showing persistent rises in maternal fruit consumption. In contrast, shorter-term studies (≤1 year postpartum), such as Nasuti et al. (19) (Canada), Poulain et al. (20) (Germany), and Hartmann et al. (18) (Switzerland), report inconsistent effects – ranging from positive to null or even negative.

In the UK sample, however, we observe no significant differences in fruit and vegetable consumption between parents and childless individuals also in the long-term. This is an unexpected result given the patterns in other liberal welfare states. Potential explanations for this unexpected finding are given below.

The existing literature sufficiently demonstrates that the transition to parenthood can influence parents’ fruit and vegetable consumption behavior. Our study adds to this finding with the important observation that the effect is not universal, but can vary depending on national context, such as differences in childcare and existing social norms – gender and education. Most of the influence appears to be positive. A more general explanation for this effect is parents’ sense of responsibility for their children’s nutrition and their role as role models. However, increased stress and unfavorable coping mechanisms related to eating habits could potentially mitigate this effect. Specifically, gendered differences in fruit and vegetable consumption may stem from varying exposure to health-related information and societal expectations during the transition to parenthood. Women engage more with the healthcare system during pregnancy and breastfeeding, facing social pressures tied to fetal and infant health (34, 35). They also receive nutrition-related information during this period (36). A further factor that should be considered is the pursuit of weight loss after childbirth, as beauty standards regain importance after pregnancy (37). Notably, higher education groups are particularly likely to pursue this ideal (38). In contrast, men are less influenced by slimming ideals and prioritize pleasure and satiety in their dietary choices (39, 40). Because women on average spend more time on caregiving - especially time-intensive tasks such as cooking for the family - their dietary patterns may be more influenced by their role as parents. This may explain why, over time, mothers in particular are more likely to consume fruit and/or vegetables on a daily basis.

The educational stratification indicates that individuals with higher levels of education exhibit a higher frequency of daily vegetable consumption over an extended period, and they increase their daily intake of fruits and/or vegetables to a greater extent than parents with lower or medium educational attainment. The novelty of our findings lies not in the static educational gradient in fruit and vegetable consumption, but in demonstrating that both the effect of parenthood (the Average Treatment Effect on the Treated) and its timing vary significantly by educational attainment. The greater resources (time, knowledge, skills, and income) available to highly educated individuals, which are particularly relevant to vegetable consumption, may provide a rationale for this effect. For instance, higher-educated parents are more likely to have increased financial resources. Since access and affordability are critical determinants of fruit and vegetable consumption also in high-income contexts (41), higher-educated persons may have a structural advantage to access (e.g., better transport options) and afford fruit and vegetables (e.g., by ordering healthy meals) (8, 12). This suggests that they may be better positioned to engage in nutrition-related socialization efforts and benefit from these activities to a greater extent themselves, a phenomenon described as the “echo effect” (42, 43).

The observation that these effects were only significant in Australia and not in the UK could be an indication of the influence of cultural differences. A comparative qualitative study by Aschemann-Witzel and Moura (44) underscores the significance of cultural variations in parents’ perceptions of changes in dietary habits during the transition to parenthood. For instance, disparities in perceptions of social support (e.g., family, friends, health professionals), enjoyment of food preparation, parenting style, country food culture, and perceptions of health-related self-care may offer further explanations for the observed variations. Previous studies have argued along similar lines, suggesting that contextual factors may explain cross-country differences. For example, Ball et al. (45) found that the effects of socioeconomic circumstances on fruit and vegetable consumption were not consistent across countries, potentially due to such contextual influences. Even though we selected relatively similar countries, there are some differences that might matter for the effect of parenthood on fruit and vegetable consumption. In Australia, net childcare costs, including rent subsidies, are significantly lower than in the UK. During the observation period of this study (2007–2018), this discrepancy reached 12 percentage points (46). Furthermore, Australia exhibits higher rates of formal childcare enrollment compared to the UK (47). The combination of reduced childcare expenditures and enhanced access to formal childcare may result in increased available financial resources and less time strain for Australian parents, which could help explain the observed disparities between the two countries.

The differential effect of parenthood on fruit and vegetable consumption, however, could also be due to a selection effect. In Australia, people who become parents have a lower fruit and vegetable consumption than childless people, whereas in the UK the pre-parenthood group already has higher fruit and vegetable consumption than childless persons. Thus, there is positive selection into parenthood in the UK in terms of fruit and vegetable consumption, which may reduce the likelihood of parenthood to further increase healthy eating. As a result, baseline levels of fruit and vegetable consumption of parents in the UK are higher than in Australia leaving less room for improvement. Future research could further explore the influence of such selection mechanisms by employing instrumental variables or by using pooled OLS general trends regression model – which assume that changes in the outcome do not follow a parallel trend before treatment – but this approach was not feasible given the number of waves and observations in our data (48). Also, country-difference in social desirability of fruit and vegetable consumption could be explored as an explanation for the observed differences.

### 4.1 Limitations

It is crucial to acknowledge the limitations of this study and contextualize the findings. The consumption of fruit and vegetables constitutes only a part of a healthy dietary regime. Evidence suggests that both positive and negative dietary behaviors become more prevalent with the onset of parenthood (16, 19, 49). Our focus on fruit and vegetable consumption is therefore a limited measure of healthy eating and the impact of parenthood on dietary behavior. Moreover, our harmonized measure based on general household surveys, is relatively simple as it only indicates the frequency of consumption and not the quantity or quality of the fruit and vegetables that were consumed. However, as the primary focus of our study is on changes in dietary patterns between parents and childless people based on harmonized dichotomous variables, we consider this approach to be a good starting point, especially as a significant proportion of the population does not report consuming any amount of fruit and vegetables on a daily basis.

As our study is based on self-reported dietary data. The findings may be subject to reporting bias, particularly the overreporting of healthy foods such as fruits and vegetables due to social desirability (50, 51). Such biases may differ by gender, with some studies suggesting that women show stronger associations between social desirability or social approval and reported diet quality, possibly due to greater concern with health and body image (52–54). However, most existing studies on gender differences in reporting bias focus on total energy or macronutrient intake rather than on fruit and vegetable consumption specifically, and further research is needed in this area. Evidence regarding education is mixed. While some studies suggest a stronger bias among individuals with lower educational attainment, others report no significant association (51, 55).

While the survey instruments used in the UKHLS and HILDA datasets are similar, it is important to recognize that the definition of vegetables differs slightly. Australian guidelines classify potatoes as vegetables, whereas the UK explicitly excludes them from this category. In the HILDA dataset, the definition of vegetables is not restricted in this manner, meaning that potatoes (not including chips or French fries) are included as a vegetable portion. In contrast, the UKHLS survey applies a definition consistent with UK guidelines, which explicitly excludes potatoes from the vegetable category (56, 57). This difference may slightly affect the overall levels of reported vegetable consumption and should be considered when interpreting cross-country differences. However, similar patterns in fruit consumption - where no definitional discrepancies exist - suggest that this factor alone is unlikely to account for the observed results.

Another limitation arises from the modest number of cases in the transitions to parenthood which meant that we had to use a limited number of control variables in our models and could not differentiate between men and women in the models stratified by education. Moreover, sensitivity analyses with parts of the sample or alternative specifications could not be conducted.

Nevertheless, our fixed effects models do already take into account time-constant group differences. Other relevant variables that could act as a mediators between parenthood and fruit and vegetable consumption such as employment and further children were intentionally excluded from our models since they are likely to be endogenous to the birth of the first child.

In addition to the statistical associations we report, it is important to situate these findings within the everyday contexts of parenthood. Parents’ fruit and vegetable provisioning occurs under conditions shaped by time constraints, household resources, caregiving responsibilities, and local food environments. Our data do not fully capture the meanings parents attribute to these practices or the situational complexities of family life; therefore, the results should be interpreted with these contextual limitations in mind.

Finally, our comparative analysis was limited to two countries which was due to the lack of panel data from other countries that could be harmonized. With this limited number of country cases, the effects of institutional and cultural factors could not be estimated in our models. If in the future, comparative data for a sizeable number of countries become available, this would substantially enhance our understanding of the country differences in our findings. Moreover, the countries in our study are from a high-income context and thus results can likely not be generalized to low- and middle-income countries (LMIC) where gender inequalities as well as practical and financial obstacles (58) contribute to a lower fruit and vegetable intake (59). A small number of studies has explored the effect of the number of children on fruit and vegetable consumption of parents in LMICs with mixed results (60).

## 5 Conclusion

This study highlights that parenthood can positively influence parents’ fruit and vegetable consumption. While most parents recognize that a diet rich in fruit and vegetables is essential for good health – a finding supported by numerous qualitative studies (8, 43, 61) - many struggle to incorporate and maintain these healthy eating habits consistently in their daily life. Given the significant implications for both parental and child health, it is essential to provide targeted support to help parents sustain higher fruit and vegetable intake. Our analysis underscores the need to consider various factors, including the child’s age, parental gender, education level, and broader cultural and national contexts, when designing such interventions. To deepen our understanding of parental dietary behaviors, future comparative panel studies are necessary.

## Data Availability

This study analyzed publicly available datasets. Specifically, two independent longitudinal household surveys were used. The Australian data came from the Household, Income and Labour Dynamics in Australia (HILDA) Survey. This survey is provided by the Department of Social Services and the Melbourne Institute of Applied Economic and Social Research. The data is from General Release 21 (Waves 1–21) and is available at https://doi.org/10.26193/KXNEBO. The UK data come from the British Household Panel Survey (BHPS) and are available via the UK Data Service at https://beta.ukdataservice.ac.uk/datacatalogue/studies/study?id=6614, DOI: 10.5255/UKDA-SN-6614-20.
