# Supplementary material for: Changes in fruit and vegetable consumption during the transition to parenthood: longitudinal evidence from Australia and the United Kingdom
Source: Front Public Health. 2026 Jan 13;13:1673209. doi: 10.3389/fpubh.2025.1673209 (PMC12834806; doi:10.3389/fpubh.2025.1673209)
Supplement: Supplementary file 1 [file Data_Sheet_1.pdf]

Table A1. Overview of longitudinal studies on the effects of parenthood on fruit and vegetable consumption

Study	Country	Operationalization of fruit and vegetable consumption	DID/FE[a] Design	Observation period and times; reference group	Participants	Results	
						Having a child	Remain childless
Tracking of Food Choices across the Transition to Motherhood (*Olson et al. 2005*)	USA	Portions of fruit and vegetables a day	No	Approx. 2.5 years (1995 - 1997); t 0-5 (before pregnancy to 2 years after) Women who had no children at t0 and were not pregnant	360 women (160 expectant mothers) from a geographically restricted sample	Fruit and Vegetable ↗*	-
Transitions in Living Arrangements Are Associated with Changes in Dietary Patterns in Young Women (*Elstgeest et al. 2012*)	Australia	Portions of fruit and vegetables a day	No	6 years (t0: 2003-t1: 2009); Women who had no children at t0	6534 (including 2484 expectant mothers) women from national random sample (cohorts 1973-1978)	(cooked) vegetables ↗* fruit ↗*	(cooked) vegetables → fruit ↗* (No statistical test for difference)
Changes in Diet Behavior when Adults Become Parents (*Laroche et al. 2012*)	USA	Portions of fruit and vegetables a day	No	7 years (t0: 1985/86 - t1: 1992/93); People who had no children at t0	2563 (including 808 parents-to-be) from a geographically and ethnically restricted sample	Fruit and Vegetable ↗	Fruit and Vegetable ↗ (Test for difference, not significant)
Time for change? Food choices in the transition to cohabitation	Switzerland	Portions of fruit and vegetables a day	No	1 year (t0 and t1: 2010-2011 or 2011-2012); People who had no children in the household at t0	3559 (including 65 first-time parents, 37 men and 28 women) from household survey	Vegetable ↗* Fruit → (Change in vegetable consumption for women only)	-

and parenthood (*Hartmann et al. 2014*)							
Comparison of the Dietary Intakes of New Parents, Second-Time Parents, and Nonparents: A Longitudinal Cohort Study (*Nasuti et al. (2014*)	Canada	Portions of fruit and vegetables a day	No	Approx. 1 year (between 2007 and 2011); t 0-2 (during pregnancy to one year after birth); pregnant women at t0	334 people (including 138 parents-to-be) from a non-random and geographically restricted sample	Fruit ↘*<br>Vegetable →	Fruit →<br>Vegetable →
Changes in diet from pregnancy to one year after birth: a longitudinal study (*Poulain et al. 2021*)	Germany	Portions of fruit and vegetables a day	No	Approx. 1 year (between 2016 and 2020); t 0-2 (during pregnancy to one year after birth)	110 women (71 of whom gave birth for the first time) from a non-random and geographically restricted sample	Fruit and Vegetable ↘* (Note: Applies to first-time and multiparous women. No significant difference in diet between first-time and multiparous women).	-

Note: a: Difference-in-Difference/Fixed-Effect; * Statistically significant

Table A2: Differences in the Proportion of Individuals with Daily Consumption of Vegetables, Fruits, or Either (Vegetables and/or Fruits) Across Time Points Before and After Childbirth in Australia and the UK, Stratified by Gender

	Men						Women					
	Veg. and/or Fr.	Veg.	Fr.	Veg. and/or Fr.	Veg.	Fr.	Veg. and/or Fr.	Veg.	Fr.	Veg. and/or Fr.	Veg.	Fr.
**Country**		AU			UK			AU			UK	
**Time before / since birth (Ref.: BY-3a+)**												
BY-1/2a	0.019	0.034	-0.001	0.003	0.042	-0.043	0.069***	0.043	0.036	-0.041*	-0.051**	-0.020
	(0.029)	(0.029)	(0.024)	(0.028)	(0.028)	(0.027)	(0.026)	(0.027)	(0.027)	(0.023)	(0.023)	(0.024)
BY+1a	0.064**	0.057*	0.040	0.010	0.051*	0.004	0.100***	0.065**	0.102***	0.000	-0.020	0.040
	(0.030)	(0.029)	(0.026)	(0.031)	(0.030)	(0.028)	(0.026)	(0.027)	(0.026)	(0.023)	(0.023)	(0.025)
BY+2/3a	0.075**	0.064*	0.048	0.006	0.076**	-0.045	0.067**	0.051	0.129***	-0.039	-0.028	-0.012
	(0.035)	(0.035)	(0.031)	(0.034)	(0.034)	(0.032)	(0.032)	(0.033)	(0.033)	(0.027)	(0.027)	(0.028)
BY+4/5a	0.065*	0.051	0.041	-0.000	0.062	-0.010	0.127***	0.057	0.123***	-0.042	-0.022	-0.037
	(0.040)	(0.040)	(0.036)	(0.042)	(0.041)	(0.039)	(0.035)	(0.037)	(0.037)	(0.032)	(0.033)	(0.033)
BY+6a+	0.131***	0.082*	0.112***	-0.028	0.053	-0.042	0.059	0.027	0.120***	0.020	0.021	-0.019
	(0.042)	(0.043)	(0.038)	(0.057)	(0.057)	(0.052)	(0.040)	(0.042)	(0.042)	(0.046)	(0.047)	(0.046)
**Education (Ref.: Low)**												
high	0.128***	0.122***	0.086***	0.138***	0.137***	0.109***	0.166***	0.178***	0.121***	0.153***	0.166***	0.128***
	(0.018)	(0.017)	(0.016)	(0.013)	(0.013)	(0.012)	(0.017)	(0.018)	(0.017)	(0.011)	(0.012)	(0.011)
**Age**	-0.001	-0.003	0.002	0.005**	0.001	0.007***	0.007***	0.008***	0.002	0.006***	0.004**	0.007***
	(0.003)	(0.003)	(0.002)	(0.002)	(0.002)	(0.002)	(0.003)	(0.003)	(0.003)	(0.002)	(0.002)	(0.002)
**Partner (Ref.: No partner)**												
Partner	0.038**	0.060***	-0.009	0.064***	0.057***	0.026*	-0.006	-0.007	-0.031*	0.076***	0.075***	0.065***
	(0.017)	(0.017)	(0.015)	(0.014)	(0.014)	(0.013)	(0.017)	(0.017)	(0.017)	(0.012)	(0.012)	(0.011)
**Birth cohort (Ref.: 1970-1979)**												
1980-1991	0.005	-0.017	0.019	0.037	0.001	0.055**	0.024	0.050	-0.014	0.007	0.009	0.039*

	(0.033)	(0.032)	(0.029)	(0.026)	(0.027)	(0.024)	(0.033)	(0.036)	(0.034)	(0.023)	(0.024)	(0.023)
**Wave - main year of data collection**												
2007		REF.		N/A	N/A	N/A		REF.		N/A	N/A	N/A
2009	-0.004	-0.025	0.008	N/A	N/A	N/A	-0.022	-0.024	0.005	N/A	N/A	N/A
	(0.017)	(0.016)	(0.014)				(0.017)	(0.017)	(0.016)			
2011	N/A	N/A	N/A		REF.		N/A	N/A	N/A		REF.	
2013	0.047**	0.030	0.019	N/A	N/A	N/A	0.013	0.015	0.024	N/A	N/A	N/A
	(0.023)	(0.022)	(0.020)				(0.023)	(0.023)	(0.022)			
2014	N/A	N/A	N/A	0.029**	0.029**	0.015	N/A	N/A	N/A	0.012	0.010	-0.015
				(0.013)	(0.013)	(0.012)				(0.011)	(0.011)	(0.012)
2016	N/A	N/A	N/A	-0.009	-0.001	-0.036**	N/A	N/A	N/A	0.008	-0.005	-0.031**
				(0.016)	(0.016)	(0.015)				(0.014)	(0.014)	(0.014)
2017	0.043	0.030	0.001	N/A	N/A	N/A	-0.008	0.006	0.017	N/A	N/A	N/A
	(0.032)	(0.030)	(0.028)				(0.032)	(0.033)	(0.032)			
2018	N/A	N/A	N/A	-0.074***	-0.058***	-0.093***	N/A	N/A	N/A	-0.080***	-0.063***	-0.113***
				(0.020)	(0.020)	(0.018)				(0.017)	(0.017)	(0.017)
**Ever parent (GFE)**	-0.035	-0.047*	-0.009	-0.003	-0.043	0.031	-0.064***	-0.074***	-0.016	0.048**	0.043*	0.043*
	(0.025)	(0.025)	(0.021)	(0.027)	(0.026)	(0.025)	(0.024)	(0.024)	(0.023)	(0.021)	(0.022)	(0.022)
**Constant**	0.401***	0.398***	0.145*	0.211***	0.268***	0.006	0.336***	0.204**	0.266***	0.263***	0.204***	0.054
	(0.089)	(0.087)	(0.079)	(0.072)	(0.073)	(0.067)	(0.087)	(0.092)	(0.089)	(0.062)	(0.065)	(0.062)
**Observations**	6056	6056	6056	9906	9906	9906	5746	5746	5746	13159	13159	13159

Source: Harmonized dataset based on HILDA (Australia) and UKHLS (UK) panel data, N = 34,867 person-years. Own calculations and visualization. All estimates are derived from Pooled Ordinary Least Squares Models with Group Fixed Effects (POLS-GFE). The dependent variable is daily consumption of vegetables, fruits, or either (vegetables and/or fruits), stratified by country and gender. Coefficients (with robust standard errors in parentheses) represent the estimated difference in the proportion of daily consumption between parents and the same individuals had they remained childless (counterfactual comparison). REF.: Reference category. N/A: Not available due to missing data. * $p < 0.10$, ** $p < 0.05$, *** $p < 0.01$.

Table A3: Differences in the Proportion of Individuals with Daily Consumption of Vegetables, Fruits, or Either (Vegetables and/or Fruits) Across Time Points Before and After Childbirth in Australia and the UK, Stratified by Educational Group

	**Vegetable and/or Fruit Consumption**				**Vegetable Consumption**				**Fruit Consumption**			
**Country**	AU		UK		AU		UK		AU		UK	
**Education Status**	Low/Mid.	High	Low/Mid.	High	Low/Mid.	High	Low/Mid.	High	Low/Mid.	High	Low/Mid.	High
**Time before / since birth (Ref.: BY-3a+)**												
BY-1/2a	0.025	0.071**	-0.043	-0.008	0.011	0.073**	-0.031	-0.000	-0.008	0.056*	-0.034	-0.026
	(0.026)	(0.029)	(0.028)	(0.024)	(0.026)	(0.031)	(0.027)	(0.024)	(0.022)	(0.029)	(0.026)	(0.025)
BY+1a	0.083***	0.087***	0.005	0.001	0.066**	0.060**	0.012	0.004	0.051**	0.102***	0.020	0.028
	(0.028)	(0.029)	(0.030)	(0.024)	(0.027)	(0.029)	(0.028)	(0.024)	(0.025)	(0.029)	(0.028)	(0.025)
BY+2/3a	0.065*	0.082**	-0.049	-0.002	0.035	0.086**	0.007	0.017	0.056*	0.134***	-0.062**	0.002
	(0.033)	(0.034)	(0.033)	(0.027)	(0.033)	(0.036)	(0.032)	(0.028)	(0.030)	(0.035)	(0.030)	(0.029)
BY+4/5a	0.086**	0.115***	-0.035	-0.021	0.017	0.096**	0.039	-0.013	0.087**	0.088**	-0.055	-0.003
	(0.037)	(0.037)	(0.039)	(0.034)	(0.037)	(0.040)	(0.039)	(0.035)	(0.035)	(0.039)	(0.036)	(0.035)
BY+6a+	0.088**	0.109***	0.044	-0.032	0.049	0.066	0.084	-0.001	0.085**	0.157***	0.011	-0.053
	(0.040)	(0.041)	(0.057)	(0.046)	(0.040)	(0.044)	(0.057)	(0.047)	(0.037)	(0.044)	(0.053)	(0.046)
**Female**	0.115***	0.168***	0.077***	0.092***	0.090***	0.164***	0.058***	0.087***	0.108***	0.147***	0.081***	0.102***
	(0.016)	(0.017)	(0.012)	(0.012)	(0.016)	(0.018)	(0.012)	(0.013)	(0.015)	(0.017)	(0.011)	(0.012)
**Age**	0.004	0.003	0.002	0.009***	0.003	0.004	0.000	0.006***	0.005*	-0.001	0.004**	0.010***
	(0.003)	(0.003)	(0.002)	(0.002)	(0.002)	(0.003)	(0.002)	(0.002)	(0.002)	(0.003)	(0.002)	(0.002)
**Partner status (Ref.: No partner)**												
Partner	0.001	0.044**	0.063***	0.077***	0.018	0.049***	0.049***	0.084***	-0.026*	-0.013	0.041***	0.056***
	(0.016)	(0.018)	(0.013)	(0.013)	(0.015)	(0.019)	(0.012)	(0.013)	(0.014)	(0.019)	(0.012)	(0.013)
**Birth cohort (Ref.: 1970-1979)**												
1980-1991	0.032	0.003	-0.031	0.067***	0.042	-0.003	-0.028	0.038	0.046	-0.035	-0.001	0.088***

	(0.034)	(0.032)	(0.025)	(0.023)	(0.033)	(0.034)	(0.025)	(0.025)	(0.030)	(0.033)	(0.023)	(0.024)
**Wave - main year of data collection**												
2007	0.000	0.000	N/A	N/A	0.000	0.000	N/A	N/A	0.000	0.000	N/A	N/A
	(.)	(.)			(.)	(.)			(.)	(.)		
2009	-0.027*	0.014	N/A	N/A	-0.043***	0.006	N/A	N/A	0.006	0.008	N/A	N/A
	(0.015)	(0.020)			(0.015)	(0.020)			(0.013)	(0.020)		
2011	N/A	N/A	0.000	0.000	N/A	N/A	0.000	0.000	N/A	N/A	0.000	0.000
			(.)	(.)			(.)	(.)			(.)	(.)
2013	0.017	0.052**	N/A	N/A	0.000	0.059**	N/A	N/A	0.009	0.040	N/A	N/A
	(0.021)	(0.025)			(0.021)	(0.026)			(0.019)	(0.025)		
2014	N/A	N/A	0.030**	0.013	N/A	N/A	0.026**	0.016	N/A	N/A	0.004	-0.004
			(0.013)	(0.012)			(0.012)	(0.012)			(0.012)	(0.012)
2016	N/A	N/A	0.013	-0.008	N/A	N/A	-0.000	-0.005	N/A	N/A	-0.007	-0.054***
			(0.016)	(0.014)			(0.015)	(0.015)			(0.014)	(0.015)
2017	0.004	0.036	N/A	N/A	-0.013	0.055	N/A	N/A	-0.006	0.031	N/A	N/A
	(0.030)	(0.033)			(0.030)	(0.035)			(0.027)	(0.034)		
2018	N/A	N/A	-0.049***	-0.100***	N/A	N/A	-0.040**	-0.078***	N/A	N/A	-0.069***	-0.133***
			(0.019)	(0.017)			(0.018)	(0.018)			(0.017)	(0.018)
**Ever parent (GFE)**	-0.037*	-0.070***	0.031	0.019	-0.041*	-0.089***	0.010	-0.000	-0.008	-0.024	0.039*	0.029
	(0.022)	(0.026)	(0.025)	(0.022)	(0.022)	(0.027)	(0.025)	(0.023)	(0.019)	(0.026)	(0.023)	(0.023)
**Constant**	0.298***	0.406***	0.351***	0.187***	0.245***	0.311***	0.317***	0.216***	0.065	0.327***	0.124**	-0.023
	(0.086)	(0.093)	(0.067)	(0.066)	(0.083)	(0.101)	(0.067)	(0.071)	(0.077)	(0.097)	(0.062)	(0.068)
**Observations**	6652	5150	11267	11798	6652	5150	11267	11798	6652	5150	11267	11798

Source: Harmonized dataset based on HILDA (Australia) and UKHLS (UK) panel data, N = 34,867 person-years. Own calculations and visualization. All estimates are derived from Pooled Ordinary Least Squares Models with Group Fixed Effects (POLS-GFE). The dependent variable is daily consumption of vegetables, fruits, or either (vegetables and/or fruits), stratified by country and education. Coefficients (with robust standard errors in parentheses) represent the estimated difference in the proportion of daily consumption between parents and the same individuals had they remained childless (counterfactual comparison). REF.: Reference category. N/A: Not available due to missing data. $^{*}p < 0.10$, $^{**}p < 0.05$, $^{***}p < 0.01$.

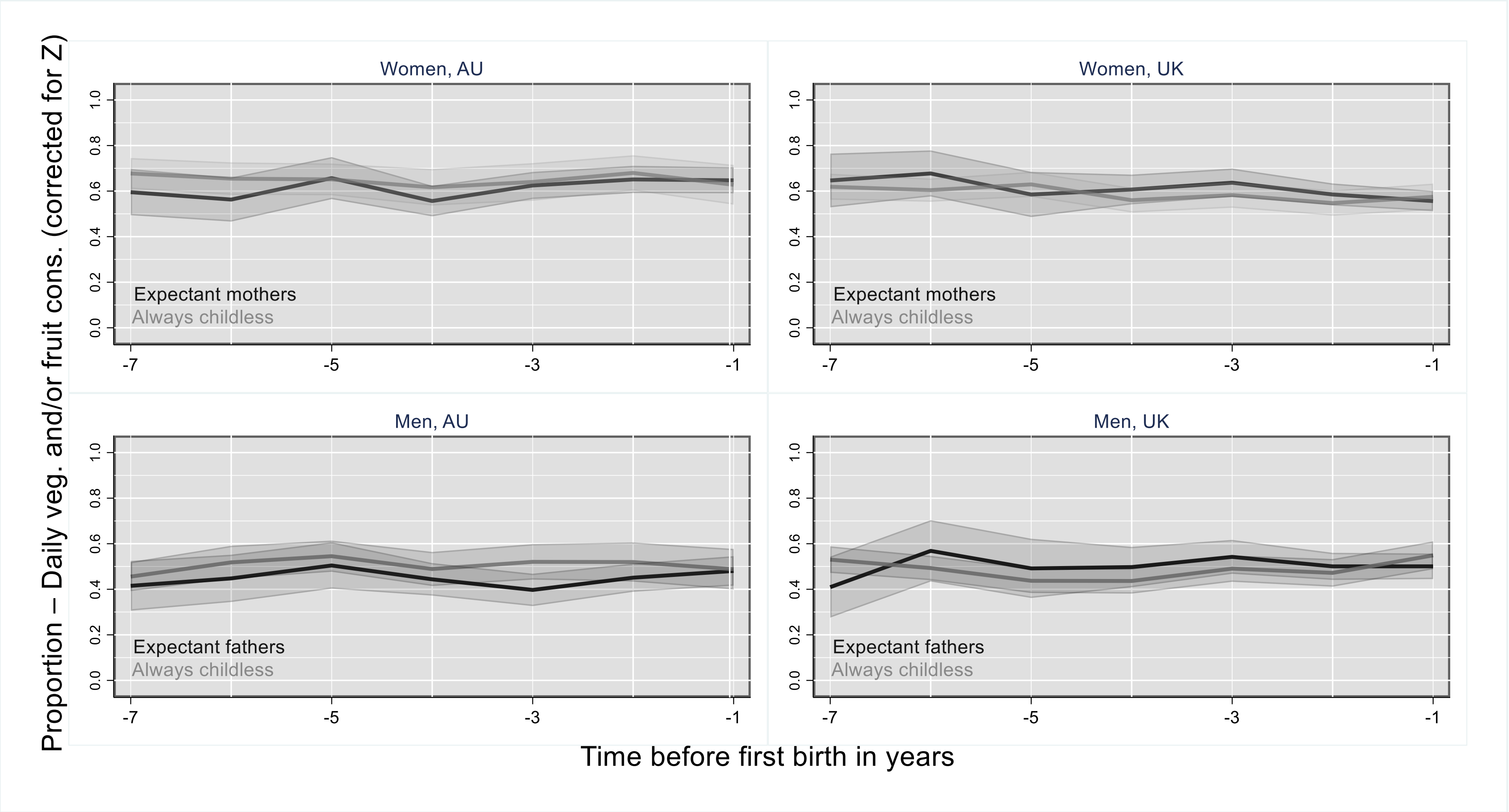

Figure A1: Proportion of daily fruit and/or vegetable consumption before first birth by sex and country. Notes: Probabilities adjusted for age, education, relationship status, birth cohort, and survey year. Shaded areas represent 95% confidence intervals. Harmonized dataset based on HILDA and UKHLS panel data, N= 34,867 person-years.

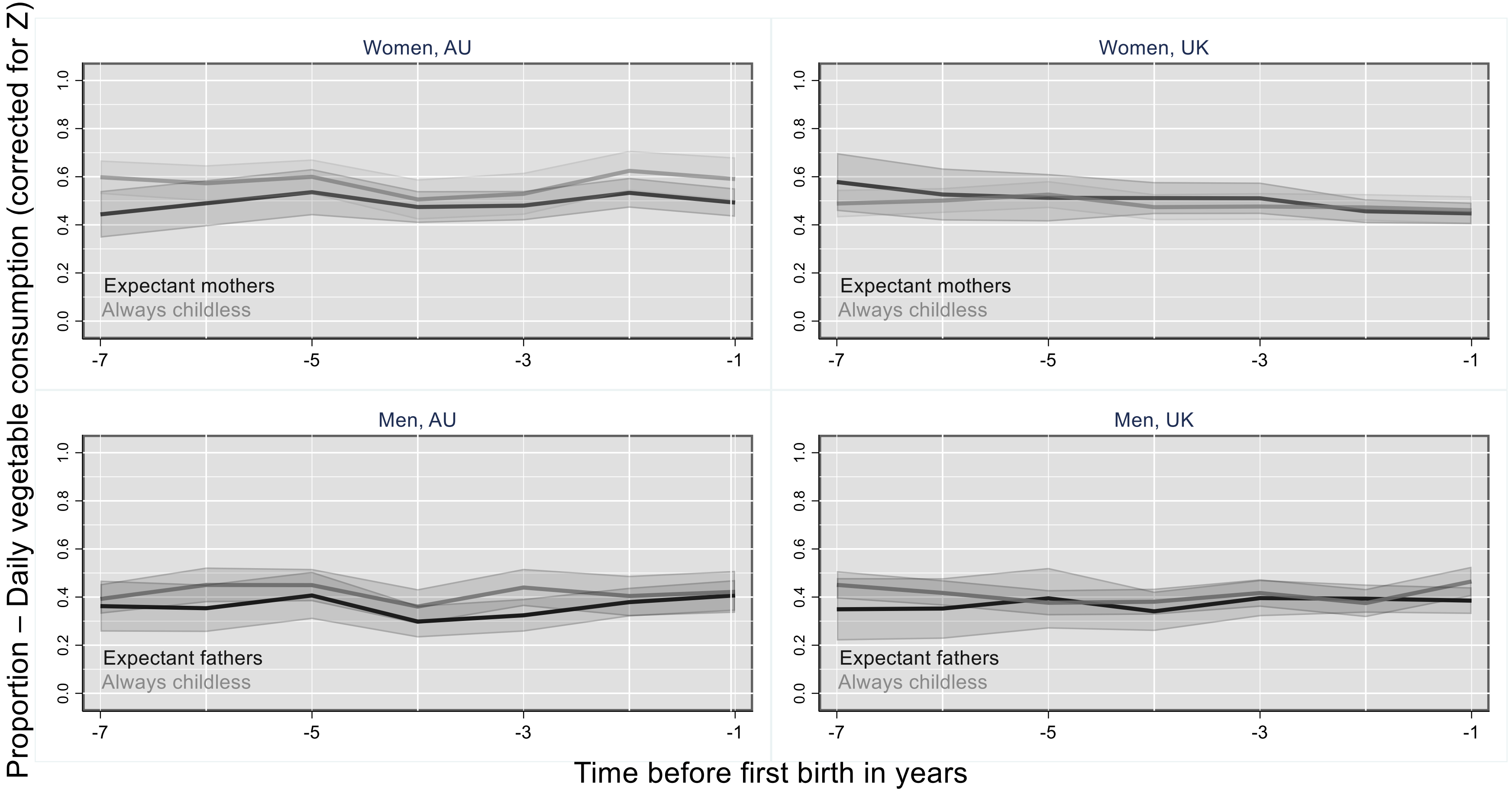

Figure A2: Proportion of daily vegetable consumption before first birth by sex and country. Notes: Probabilities adjusted for age, education, relationship status, birth cohort, and survey year. Shaded areas represent 95% confidence intervals. Harmonized dataset based on HILDA and UKHLS panel data, N= 34,867 person-years.

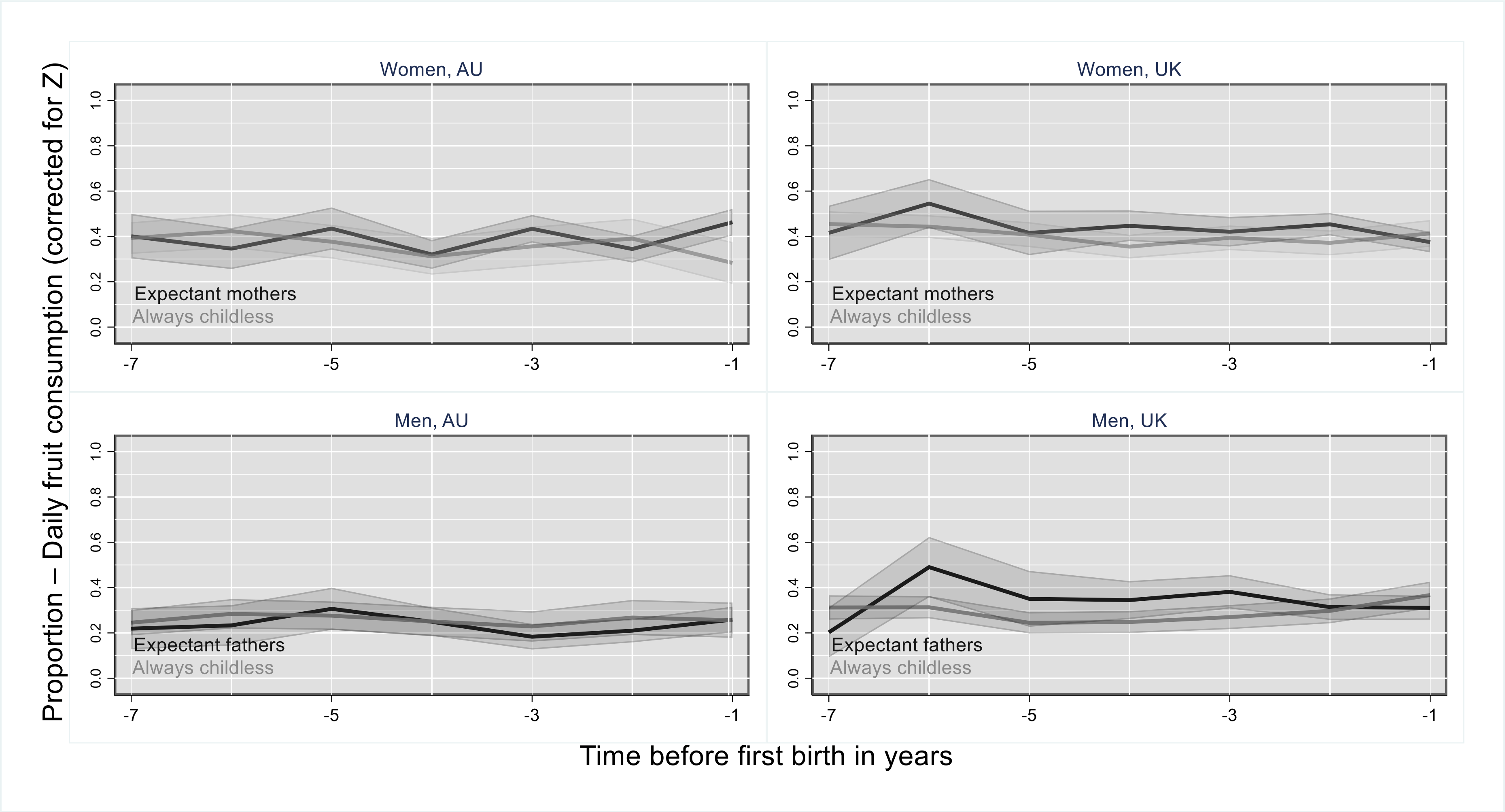

Figure A2: Proportion of daily fruit consumption before first birth by sex and country. Notes: Probabilities adjusted for age, education, relationship status, birth cohort, and survey year. Shaded areas represent 95% confidence intervals. Harmonized dataset based on HILDA and UKHLS panel data, N= 34,867 person-years.
